# Supplementary material for: How good are pathogenicity predictors in detecting benign variants?
Source: PLoS Comput Biol. 2019 Feb 11;15(2):e1006481. doi: 10.1371/journal.pcbi.1006481 (PMC6386394; doi:10.1371/journal.pcbi.1006481)
Supplement: S1 Fig — (A) Sensitivities and specificities of CADD at different cutoffs of phred-like scores. The authors recommend using a phred-like score between 10 to 20 for distinguishing pathogenic and benign variants. Sensitivities (black) are calculated for 1301 pathogenic and likely pathogenic variants from ClinVar. The pathogenic variants in training datasets of tools could not be excluded. Specificities (grey) are calculated for 20602 variants with adjusted allele frequencies (AF Adj) between 1% to 25% obtained from ExAC. (B) Sensitivities and specificities of VEST at different cutoffs of VEST score. (C) Sensitivity and specificity for all the tested variant interpretation tools. (D) Sensitivity and specificity for variants that were predicted by all the methods. Variants that could not be predicted by any of the tools were excluded. The number of pathogenic variants was 480 and of neutral variants was 7268. The numbers of cases were normalized prior to calculation of sensitivity and specificity. (DOCX) [file pcbi.1006481.s001.docx]

**A B**

**C
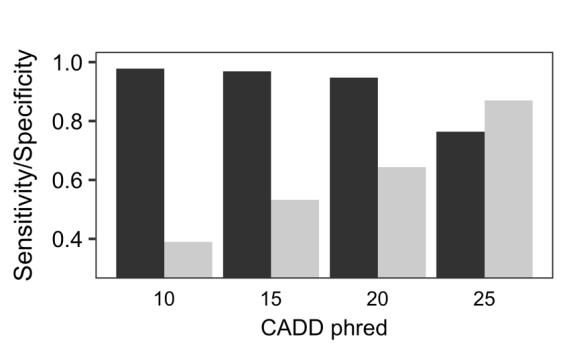

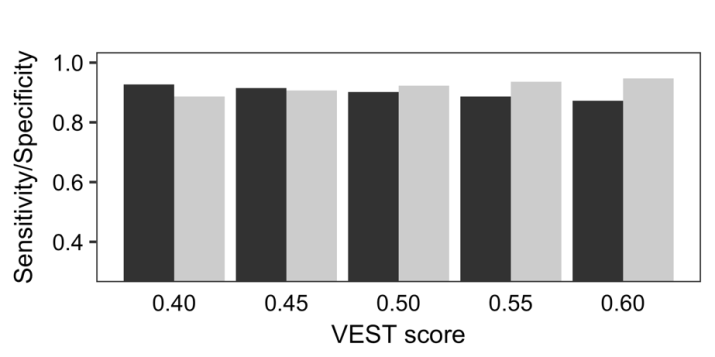
**

**
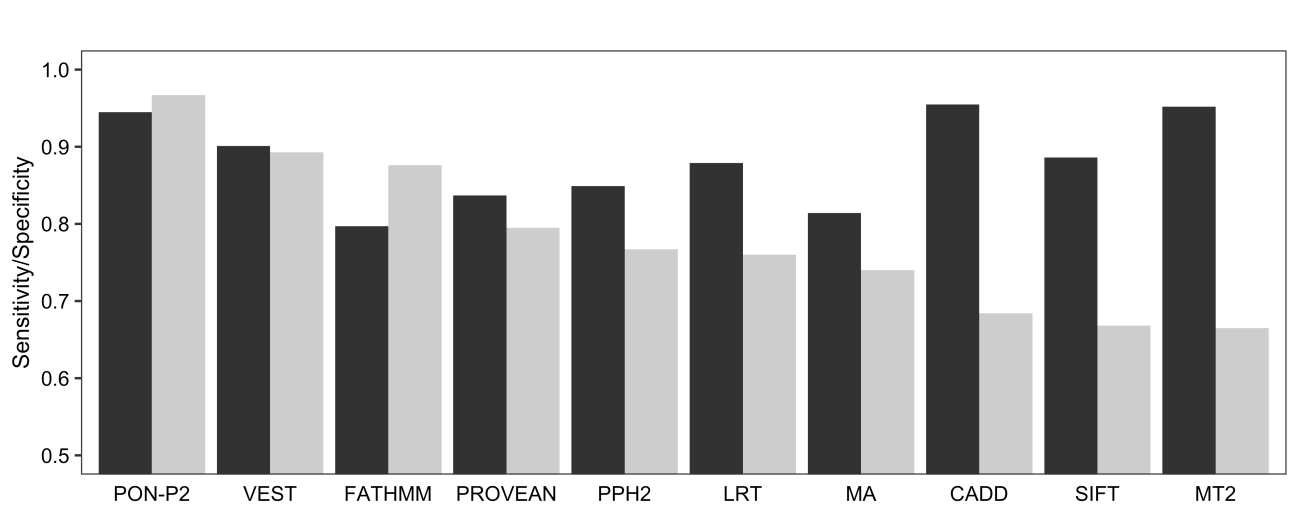
**

**D**


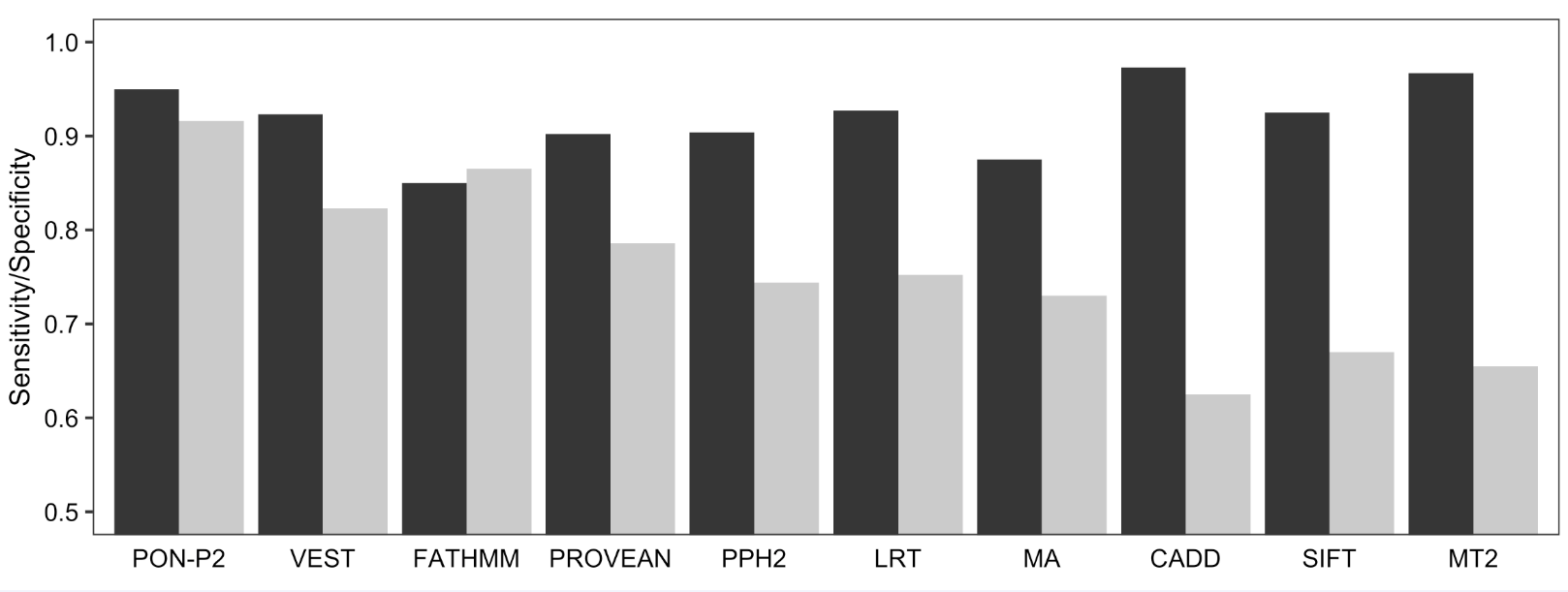


**S1 Figure. Sensitivities and specificities of the tested predictors.**

(A) Sensitivities and specificities of CADD at different cutoffs of phred-like scores. The authors recommend using a phred-like score between 10 to 20 for distinguishing pathogenic and benign variants. Sensitivities (black) are calculated for 1301 pathogenic and likely pathogenic variants from ClinVar. The pathogenic variants in training datasets of tools could not be excluded. Specificities (grey) are calculated for 20602 variants with adjusted allele frequencies (AF Adj) between 1% to 25% obtained from ExAC. (B) Sensitivities and specificities of VEST at different cutoffs of VEST score. (C) Sensitivity and specificity for all the tested variant interpretation tools. (D) Sensitivity and specificity for variants that were predicted by all the methods. Variants that could not be predicted by any of the tools were excluded. The number of pathogenic variants was 480 and of neutral variants was 7268. The numbers of cases were normalized prior to calculation of sensitivity and specificity.
