# Supplementary material for: How good are pathogenicity predictors in detecting benign variants?
Source: PLoS Comput Biol. 2019 Feb 11;15(2):e1006481. doi: 10.1371/journal.pcbi.1006481 (PMC6386394; doi:10.1371/journal.pcbi.1006481)
Supplement: S2 Fig — Fisher exact test was used for pairwise comparison of methods. The color coding indicates p value that ranges from 1 to 10–16, i.e. the steps indicate ten differences. (A) Comparison of all the data, and (B) variants that all the methods predicted. (DOCX) [file pcbi.1006481.s002.docx]

A


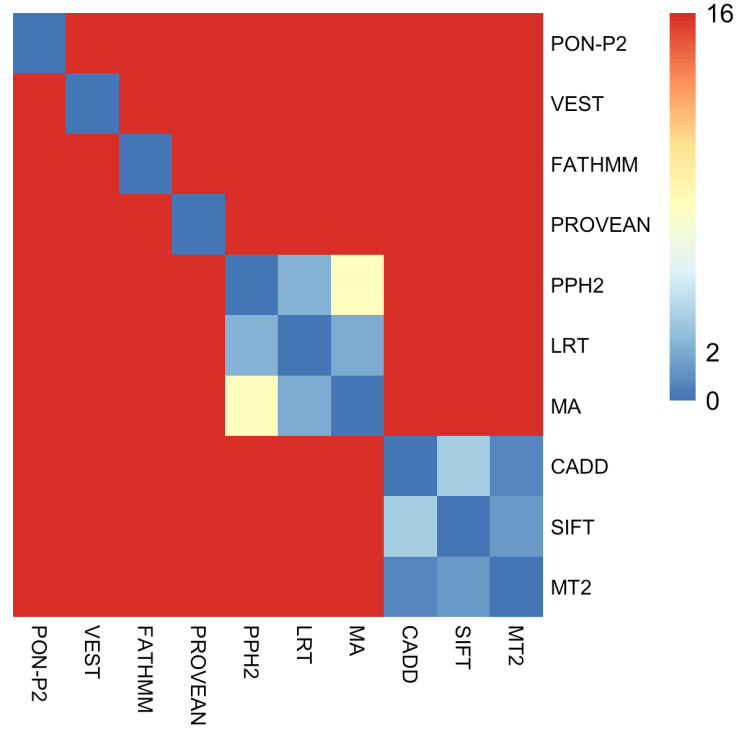


B


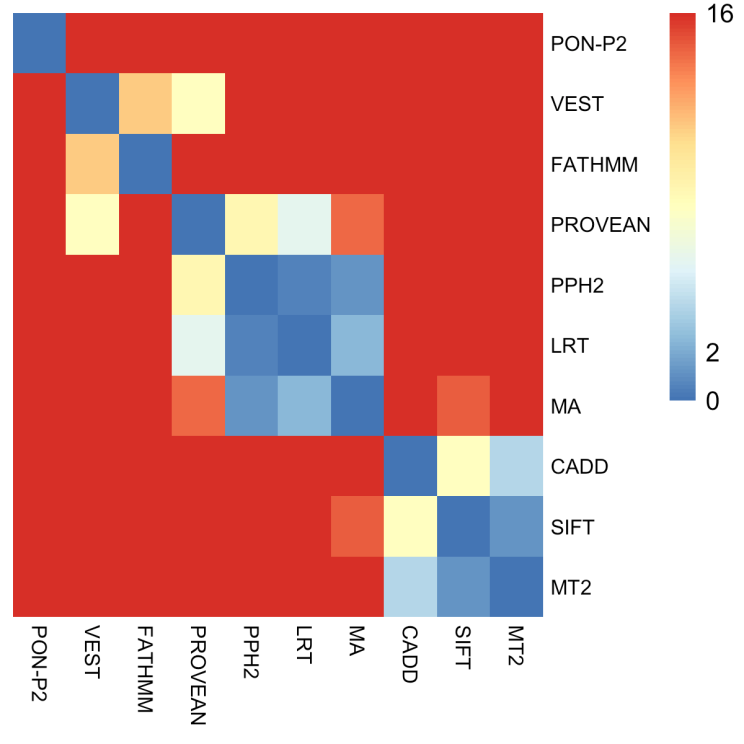


**S2 Figure. Statistical analysis of method performances.**

Fisher exact test was used for pairwise comparison of methods. The color coding indicates negative logarithm of p values that range from 0 (for p value 1) to 16 (for p value 10^-16^). All p values below 10^-16^ were set to 10^-16^. (A) Comparison of all the data, and (B) variants that all the methods predicted.
