# Supplementary material for: How good are pathogenicity predictors in detecting benign variants?
Source: PLoS Comput Biol. 2019 Feb 11;15(2):e1006481. doi: 10.1371/journal.pcbi.1006481 (PMC6386394; doi:10.1371/journal.pcbi.1006481)
Supplement: S1 Table — (DOCX) [file pcbi.1006481.s003.docx]

**S1 Table.** Percentages of variants that were not classified as pathogenic or benign.

|  | PON-P2 | FATHMM | PROVEAN | PPH2 | LRT | SIFT |
| --- | --- | --- | --- | --- | --- | --- |
| Adj | 23.0 | 0.4 | 0.8 | 2.7 | 6.0 | 1.6 |
| AFR | 24.1 | 0.4 | 0.7 | 2.6 | 6.0 | 1.7 |
| AMR | 23.2 | 0.3 | 0.8 | 2.7 | 6.2 | 1.6 |
| EAS | 24.2 | 0.4 | 0.8 | 2.6 | 6.0 | 1.7 |
| FIN | 26.9 | 0.5 | 0.8 | 2.9 | 5.7 | 1.7 |
| NFE | 24.2 | 0.4 | 0.8 | 2.7 | 6.1 | 1.7 |
| SAS | 23.7 | 0.4 | 0.7 | 2.7 | 5.9 | 1.6 |
| OTH | 23.6 | 0.4 | 0.8 | 2.7 | 5.9 | 1.7 |
