# Supplementary material for: How good are pathogenicity predictors in detecting benign variants?
Source: PLoS Comput Biol. 2019 Feb 11;15(2):e1006481. doi: 10.1371/journal.pcbi.1006481 (PMC6386394; doi:10.1371/journal.pcbi.1006481)
Supplement: S2 Table — (DOCX) [file pcbi.1006481.s004.docx]

**S2 Table.** Proportion of unique variants in the populations.

| Population | Total variants^a^ | Unique variants^b^ | Proportion of unique variants |
| --- | --- | --- | --- |
| AFR | 35,553 | 22,197 | 0.624 |
| AMR | 17,651 | 1,829 | 0.104 |
| EAS | 14,750 | 4,845 | 0.328 |
| FIN | 18,804 | 3,306 | 0.176 |
| NFE | 18,335 | 1,240 | 0.068 |
| OTH | 19,471 | 640 | 0.033 |
| SAS | 18,167 | 3,396 | 0.187 |

^a^All variants having AF ≥1% and <25% in the population.

^b^Variants having AF ≥1% and <25% in the specific population but < 1% in all other populations.
