# Supplementary material for: How good are pathogenicity predictors in detecting benign variants?
Source: PLoS Comput Biol. 2019 Feb 11;15(2):e1006481. doi: 10.1371/journal.pcbi.1006481 (PMC6386394; doi:10.1371/journal.pcbi.1006481)
Supplement: S3 Table — (DOCX) [file pcbi.1006481.s005.docx]

**S3 Table.** Specificities of tools for unique and non-unique variants in six populations. The performance scores with grey background indicate specificities for non-unique variants in the populations.

|  | AFR | AMR | EAS | FIN | NFE | SAS |
| --- | --- | --- | --- | --- | --- | --- |
| PON-P2 | 0.965 | 0.924 | 0.936 | 0.882 | 0.917 | 0.935 |
|  | 0.977 | 0.972 | 0.973 | 0.962 | 0.963 | 0.969 |
| VEST | 0.897 | 0.780 | 0.807 | 0.731 | 0.850 | 0.807 |
|  | 0.921 | 0.885 | 0.869 | 0.856 | 0.894 | 0.886 |
| FATHMM | 0.865 | 0.838 | 0.854 | 0.834 | 0.861 | 0.850 |
|  | 0.882 | 0.879 | 0.881 | 0.872 | 0.874 | 0.876 |
| PROVEAN | 0.774 | 0.741 | 0.740 | 0.697 | 0.757 | 0.743 |
|  | 0.806 | 0.800 | 0.810 | 0.785 | 0.789 | 0.799 |
| PPH2 | 0.740 | 0.675 | 0.696 | 0.618 | 0.688 | 0.699 |
|  | 0.793 | 0.777 | 0.792 | 0.754 | 0.757 | 0.772 |
| LRT | 0.743 | 0.631 | 0.650 | 0.583 | 0.644 | 0.672 |
|  | 0.789 | 0.771 | 0.789 | 0.742 | 0.746 | 0.767 |
| MA | 0.717 | 0.655 | 0.678 | 0.631 | 0.697 | 0.680 |
|  | 0.755 | 0.750 | 0.755 | 0.732 | 0.734 | 0.745 |
| CADD | 0.658 | 0.555 | 0.569 | 0.491 | 0.585 | 0.583 |
|  | 0.714 | 0.698 | 0.715 | 0.664 | 0.673 | 0.694 |
| SIFT | 0.632 | 0.556 | 0.575 | 0.552 | 0.592 | 0.589 |
|  | 0.685 | 0.677 | 0.702 | 0.671 | 0.666 | 0.681 |
| MT2 | 0.684 | 0.581 | 0.585 | 0.491 | 0.584 | 0.590 |
|  | 0.736 | 0.672 | 0.680 | 0.625 | 0.636 | 0.665 |

AFR, African; AMR, American; EAS, East Asian; FIN, Finnish; NFE, Non-Finnish European; SAS, South Asian; MA, Mutation Assessor; MT2, MutationTaster2; PPH2, PolyPhen-2.
