# Supplementary material for: How good are pathogenicity predictors in detecting benign variants?
Source: PLoS Comput Biol. 2019 Feb 11;15(2):e1006481. doi: 10.1371/journal.pcbi.1006481 (PMC6386394; doi:10.1371/journal.pcbi.1006481)
Supplement: S4 Table — (DOCX) [file pcbi.1006481.s006.docx]

**S4 Table**. Specificities of tools for unique and non-unique variants with AF ≥1% and <5% in the populations. The scores with grey background indicate specificities for the non-unique variants.

|  | AFR | AMR | EAS | FIN | NFE | SAS |
| --- | --- | --- | --- | --- | --- | --- |
| PON-P2 | 0.961 | 0.918 | 0.934 | 0.879 | 0.914 | 0.934 |
|  | 0.967 | 0.965 | 0.972 | 0.946 | 0.95 | 0.96 |
| SIFT | 0.626 | 0.551 | 0.572 | 0.551 | 0.595 | 0.59 |
|  | 0.651 | 0.65 | 0.679 | 0.637 | 0.637 | 0.646 |
| PPH2 | 0.732 | 0.674 | 0.69 | 0.616 | 0.685 | 0.697 |
|  | 0.755 | 0.753 | 0.778 | 0.722 | 0.729 | 0.74 |
| LRT | 0.729 | 0.618 | 0.645 | 0.579 | 0.639 | 0.67 |
|  | 0.74 | 0.741 | 0.767 | 0.694 | 0.702 | 0.727 |
| MT2 | 0.672 | 0.591 | 0.579 | 0.487 | 0.569 | 0.586 |
|  | 0.707 | 0.669 | 0.672 | 0.609 | 0.62 | 0.643 |
| MA | 0.707 | 0.659 | 0.675 | 0.627 | 0.692 | 0.678 |
|  | 0.732 | 0.738 | 0.748 | 0.701 | 0.705 | 0.727 |
| FATHMM | 0.864 | 0.836 | 0.853 | 0.835 | 0.863 | 0.852 |
|  | 0.879 | 0.873 | 0.875 | 0.867 | 0.864 | 0.863 |
| PROVEAN | 0.768 | 0.742 | 0.737 | 0.692 | 0.753 | 0.739 |
|  | 0.778 | 0.785 | 0.795 | 0.759 | 0.768 | 0.772 |
| CADD | 0.643 | 0.564 | 0.566 | 0.489 | 0.573 | 0.583 |
|  | 0.667 | 0.666 | 0.688 | 0.61 | 0.626 | 0.645 |
| VEST | 0.892 | 0.78 | 0.803 | 0.728 | 0.838 | 0.804 |
|  | 0.915 | 0.874 | 0.85 | 0.829 | 0.874 | 0.863 |

AFR, African; AMR, American; EAS, East Asian; FIN, Finnish; NFE, Non-Finnish European; SAS, South Asian; MA, Mutation Assessor; MT2, MutationTaster2; PPH2, PolyPhen-2.
