# Supplementary material for: How good are pathogenicity predictors in detecting benign variants?
Source: PLoS Comput Biol. 2019 Feb 11;15(2):e1006481. doi: 10.1371/journal.pcbi.1006481 (PMC6386394; doi:10.1371/journal.pcbi.1006481)
Supplement: S5 Table — (DOCX) [file pcbi.1006481.s007.docx]

**S5 Table**. Chromosome-wide numbers of variants with AF ≥1% and <25% in male and female populations.

| Chromosome | Variants in female^a^ | Variants in male | Ratio of variants in male to female |
| --- | --- | --- | --- |
| 1 | 2,301 (391) | 2,008 (103) | 0.873 (0.263) |
| 2 | 1,420 (234) | 1,268 (77) | 0.893 (0.329) |
| 3 | 1,100 (172) | 981 (54) | 0.892 (0.314) |
| 4 | 847 (147) | 754 (53) | 0.890 (0.361) |
| 5 | 882 (138) | 774 (34) | 0.878 (0.246) |
| 6 | 1,514 (174) | 1,414 (67) | 0.934 (0.385) |
| 7 | 1,039 (204) | 903 (61) | 0.869 (0.299) |
| 8 | 704 (152) | 574 (26) | 0.815 (0.171) |
| 9 | 850 (133) | 760 (40) | 0.894 (0.301) |
| 10 | 881 (126) | 785 (33) | 0.891 (0.262) |
| 11 | 1,409 (238) | 1,239 (70) | 0.879 (0.294) |
| 12 | 1,024 (157) | 907 (47) | 0.886 (0.299) |
| 13 | 316 (37) | 289 (11) | 0.915 (0.297) |
| 14 | 636 (108) | 561 (36) | 0.882 (0.333) |
| 15 | 731 (113) | 661 (44) | 0.904 (0.389) |
| 16 | 1,061 (192) | 936 (62) | 0.882 (0.323) |
| 17 | 1,191 (228) | 1,030 (59) | 0.865 (0.259) |
| 18 | 341 (56) | 302 (18) | 0.886 (0.321) |
| 19 | 1,951 (372) | 1,670 (100) | 0.856 (0.269) |
| 20 | 581 (105) | 500 (29) | 0.861 (0.276) |
| 21 | 277 (45) | 245 (12) | 0.884 (0.267) |
| 22 | 557 (92) | 488 (19) | 0.876 (0.207) |
| X | 428 (110) | 354 (31) | 0.827 (0.282) |
| Y | NA | 3 (3) | NA |
| Total | 22,041 (3724) | 19,406 (1089) | 0.880 (0.292) |
| Percentage of unique variants | 16.9 | 5.6 |  |

^a^The values in brackets are for the unique variants.
